# Supplementary figures and images for: Nerve Injury-Induced Protein 2 preserves lysosomal membrane integrity to suppress ferroptosis
Source: bioRxiv. 2026 Feb 11:2026.02.09.704867. Preprint. [Version 1] doi: 10.64898/2026.02.09.704867 (PMC12919058; doi:10.64898/2026.02.09.704867)

## Supplemental Figure 1

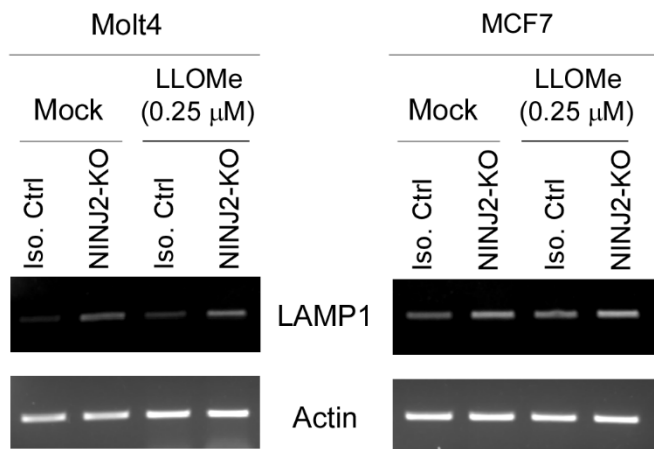

Supplement: 1 [file NIHPP2026.02.09.704867v1-supplement-1.pdf]
